# The Corneal Epithelial Thickness Profile in a Healthy Saudi Population

**DOI:** 10.7759/cureus.71135

**Published:** 2024-10-09

**Authors:** Sultan H Alrashidi

**Affiliations:** 1 Department of Ophthalmology, College of Medicine, Qassim University, Buraidah, SAU

**Keywords:** aging, anterior segment, corneal epithelial thickness, normative data, optical coherence tomography

## Abstract

Introduction: Epithelial mapping appears to be a valuable technique for a corneal and refractive surgeon, useful for distinguishing corneas with the true presence of corneal ectasia from those that are suspicious. Interpreting the epithelial thickness map data requires an understanding of corneal epithelial thickness (CET) normal values and variable patterns. Unlike corneal thickness, epithelial thickness assessment with anterior segment optical coherence tomography (AS-OCT) seems to be influenced by gender and age. The study aimed to investigate the detailed mapping of CET characteristics in normal eyes from the Saudi population and to assess its variation with age and sex using anterior segment 7-mm-wide OCT (AS-OCT) scans.

Methods: Regional epithelial thickness was assessed using an anterior radial scanning protocol with REVO NX (Optopol Technology S.A, Zawiercie, Poland) in 596 eyes of 298 individuals aged 10 to 98. CET maps in a 7 mm diameter were automatically generated by the built-in software, displaying thickness in 17 sectors divided into three zones, i) a central zone within the 0-2 mm diameter, ii) ring 1 zone, a paracentral (P-CET) zone from 2 to 5-mm, and iii) ring 2 zone, a midperipheral zone (MP-CET) from 5 to 7 mm. Ring 1 and 2 zones were further divided into eight sectors each, including superior (S), inferior (I), nasal (N), temporal (T), superonasal (SN), inferonasal (IN), superotemporal (ST), and inferotemporal (IT). An analysis was done on correlations between age and gender and the CET across different zones.

Results: Males and older adults had a substantially thicker CET than females and younger participants, with the C-CET measuring 59.2±4.5µm. In three zones, no interocular asymmetry was seen. Superiorly, CET is significantly thinner than inferiorly (p<0.05), with temporal zones being thinner than nasal zones (p<0.05). The C-CET increases with age in the seven groups of both genders, but its dependence on age is weaker in paracentral sectors; C-CET was 3.5% thicker in males. Paracentral nasal and inferior zones showed 2.2-3.6% thicker CET while the superior and temporal paracentral zones showed 3-5% thicker CET among males compared to females.

Conclusion: From 17 CET zones of central 7 mm cornea the C-CET was affected by gender and age. The CET distribution in these healthy Saudis’ eyes was non-uniform with the CET being thinner in the superior cornea. This finding could aid in predicting corneal diseases and planning refractive procedures.

## Introduction

The outermost layer of the cornea is the corneal epithelium (CE), which is composed of nonkeratinized, stratified squamous epithelium, approximately five to six cells thick, and has an average central thickness of approximately 50 to 52 μm [1,2]. The highly active, self-regenerating CE contributes significantly to the maintenance of corneal transparency and integrity. It is the only layer of the cornea that undergoes both injury-induced regeneration and maintenance with complete regeneration taking five to seven days [3]. The corneal refractive index is not uniform, and the structure of the CE at the air-tear film interface, as well as differences in refractive index with the stroma, influence the total dioptric power of the cornea. The epithelium’s refractive power varies, with an average of 1.03 D in the center 2-mm zone and 0.85 D in the 3.6-mm zone [4]. The epithelium has a non-uniform pattern, which is important to compensate for stromal thickness change to maintain the corneal thickness profile over time, create a smoother refractive surface, and preserve corneal power and ocular refraction [4,5]. In terms of regional thickness, nasal and inferior quadrants are thicker than the superior and temporal quadrants [6,7]. Conditions that alter the structure of the CE include limbal stem cell insufficiency, keratoconjunctivitis sicca, contact lens use, and corneal ectatic disorders [8], while blinking and corneal friction are thought to have a physiological influence on the corneal epithelial thickness (CET) [7].

Imaging technologies available for objectively measuring CET include optical coherence tomography (OCT), very high frequency (VHF) digital ultrasound, optical pachymetry, and confocal microscopy [1,6,7,9-13]. While confocal microscopy and VHF digital ultrasound are invasive techniques, OCT is a high-resolution, non-contact method for examining CET. Hence, SD-OCT is a widely used tool to measure CET. As clinical use of OCT continues to grow, it is increasingly important that normative databases built into OCT systems accurately represent the population to which they are applied. Mean CET is displayed at 17 points in the spectral domain OCT (SD-OCT). It was necessary to build databases of measurements taken from normal eyes to interpret these findings properly.

While there was no correlation found between gender, age, and CET in studies using SD-OCT and VHF digital ultrasonography [10,14], other investigations found that CET declined with age worldwide [10,14,15]. The mean CET for the Egyptian population was determined to be 49.2 ±2.2 mm using SD-OCT [10], whereas the mean CET for the UK population was found to be 50.7±3.7 μm using VHF digital ultrasonography [1]. These investigations concluded that there was no discernible variation in CET between males and females in any of the cornea’s zones. In contrast, the average CET of Turkish individuals was determined to be 47.8±1.15 μm, with the oldest group’s left eye CET being noticeably thinner than that of the younger groups [9]. However, an accurate understanding of the association between CET and age is still unclear.

Various studies have examined the effect of age and gender on corneal thickness in the Saudi population, however, the normative database for CET is not well established, and its association with age has not been properly established. Our study aimed to characterize CET profiles in the healthy Saudi population across a wide age range using SD-OCT. The purpose was to generate clinical normative CET data for the Saudi population and to assess if the normative data differs from similar normative CET data from other countries.

## Materials and methods

Study design and population

In this retrospective observational study, a reassessment of database records of 298 Saudi subjects from a population of individuals seeking routine eye examination at the outpatient clinic of a private tertiary care ophthalmology facility in Qassim province, Saudi Arabia between January 2022 and December 2022 was done. The research was conducted per the principles of the Declaration of Helsinki as well as local and national research standards and regulations. Although our study did not involve interventions, precautions were taken to protect the patient’s privacy and confidentiality. The study analyzed data from 596 eyes of 298 healthy Saudi males and females aged ≥10 years who had previously undergone ocular examination and OCT measurement for corneal thickness research (Alrashidi 2024)[16]. Thus, the same methodology of categorizing the subjects according to their gender and age was followed. Participants were separated into 14 groups, 7 groups for each gender based on age. Age groups include 10 to 20 years, 21 to 30 years, 31 to 40 years, 41 to 50 years, 51 to 60 years, 61 to 70 years, and more than 70 years [16].

Ocular examination

All subjects underwent a complete ocular examination, which included ocular and medical history, measurement of visual acuity using the LogMAR chart, autorefraction using the Tomey autorefractor and kerato-refractometer (Tomey Corp., Model RC-5000), and subjective refraction to determine refractive error and measurement of intraocular pressure (IOP) included with iCare tonometry, anterior and posterior segment examination. Normal subjects were those who had no complaints of eye irritation, no history of contact lens wear, no anterior segment abnormalities on biomicroscopic examination, and an apparent refractive error of ˂-4.00 diopters (D) and astigmatism of ˂-2.00 D who had the best corrected visual acuity of 20/20 or better. Subjects with eye trauma, eye surgery, corneal opacity, corneal dystrophy, keratoconus, myopia of ˃-4.00 D, history of contact lens wear, dry eye, diabetes, and autoimmune diseases were excluded from the study [16].

OCT measurement

**Figure 1:**
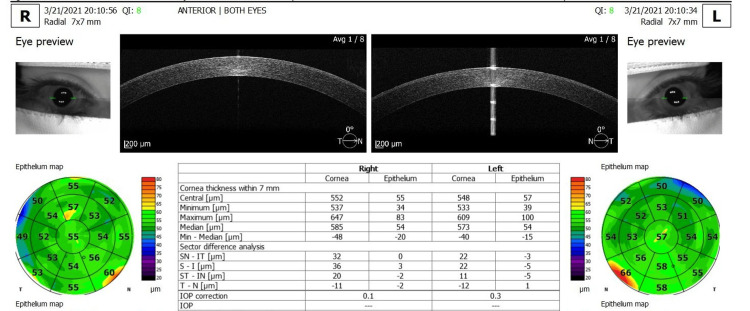
Corneal epithelial thickness maps (7 mm) of the right eye (OD) and left eye (OS) provided by the optical coherence tomography system report Source: Adapted screenshot of the output screen with the REVO FC OCT device. OCT: optical coherence tomography

The REVO FC SD-OCT device (Optopol Technology S.A, Zawiercie, Poland, software version 11.0.7) was used to acquire the corneal thickness map (CTM)/pachymetry map. It acquires corneal images using an 830 nm wavelength superluminescent diode at a scan speed of 12,000 A-scans per second and has an axial tissue resolution of 5 μm and a transverse resolution of 18 μm. All subjects’ corneas were scanned using the anterior segment radial scan protocol. The scan acquisition used to determine CET comprises eight 7-mm long radial line scans arranged at equal angles to the common axis, consisting of 2560 A-scans each, and produces a 7 mm × 7 mm single scan time pachymetry map of 1.58 seconds [17]. Each subject’s head was stabilized with a chin rest and asked to fixate on the internal fixation target on OCT. The pachymetry scans were centered on the pupil and the images were displayed on the screen in real time to facilitate alignment. If the subject moved their eyes or blinked during the measurements, the scan was repeated to obtain the required quality without artifacts [16]. Data were processed with SD-OCT review software (version 11.0), which provides the average automated CET of three concentric ring-shaped zones centered on the center of the cornea [central (C-CET): 0-2 mm, middle ring: paracentral: 2-5 mm and the outermost ring: median circumference 5-7 mm (Figure 1)]. The CET was evaluated in the sectors superior (S), superior nasal (SN), nasal (N), inferior nasal (IN), inferior (I), inferior temporal (IT), temporal (T), and superior temporal (ST) for paracentral and midperipheral sectors. Surrounding the central cornea thickness (CCT) (Zone:1) are the following paracentral sectors: paracentral S, paracentral ST, paracentral T, paracentral IT, paracentral I, paracentral IN, paracentral N and paracentral SN (Zone: 2: PC-CET: 8 sectors). Outside of these paracentral zones, there are the following midperipheral sectors: midperipheral S, midperipheral ST, midperipheral T, midperipheral IT, midperipheral I, midperipheral IN, midperipheral N, and midperipheral SN (Zone 3: MP-CET: 8 sectors). The average thickness in the different corneal epithelial sections consisting of 17 sectors was calculated and displayed accordingly in the corneal pachymetry map using a false color representation (Figure 1). The commercial software for CT mapping in the RENO OCT device determines the parameters such as CET within 7 mm (central, minimum, maximum median, and min medium thickness in m) and sectoral difference analysis (SN-IT, S-I, ST- IN, T-N in µm). The CET in seven groups of each sex was compared for age changes. The male and female groups were compared with each other in terms of the gender-specific effect [16].

Statistical analysis

Data analysis was performed with the help of using IBM Statistic SPSS (SPSS Inc., Chicago, IL, USA) version 20.0. Normality was judged via a statistical test called the Kolmogorov-Smirnov test. Continuous variables are represented by means and standard deviations, whereas differences between genders were calculated using an independent t-test. The paired t-test was performed for inter-eye comparison of the mean CET of each sector within an age group. The intraclass correlation coefficient model (ICC [1,2]) was used to measure interocular symmetry in male and female groups. The one-way ANOVA test was done to assess the variance of the mean CET of each sector across the various age groups. To assess differences between central, paracentral, and midperipheral CET, a one-way ANOVA test was used. Pearson correlation was used to correlate between age and sectoral CET. A P-value of <0.05 was considered statistically significant.

## Results

Baseline characteristics

The study included 596 eyes from 298 healthy participants, including 159 females (53.4%) and 139 men (46.6%). The average age was 41.9±20.4 years for males and 41.1±19.1 years for females. Table 1 displays the subject classification based on age and gender.

**Table 1:** Distribution of males and females according to their age group

Age Group	No. of Males (%) [Age: Mean ± SD]	No. of Females (%) [Age: Mean ± SD]	Total No. of Subjects (%) [Age: Mean ± SD]
10-20 years	23 (7.7%) [17.2±3.6]	22 (7.4%) [19.1±1]	45 (15.1%) [18.1±2.8]
21-30 years	30 (10.1%) [25.9±2.4]	42 (14.1%) [25.4±2.7]	72 (24.2%) [25.6±2.6]
31-40 years	18 (6.0%) [34.3±2.8]	23 (7.7%) [35.2±3.0]	41 (13.7%) [34.8±2.9]
41-50 years	17 (5.7%) [46.1±2.8]	23 (7.7%) [45.7±2.6]	40 (13.4%) [45.9±2.7]
51-60 years	21 (7.0%) [53.5±1.5]	19 (6.4%) [54.4±2.1]	40 (13.4%) [53.9±18.8]
61-70 years	15 (5.0%) [63.8±2.4]	15 (5.0%) [65.4±3.0]	30 (10%) [64.6±2.8]
˃ 70 years	15(5%) [86.7±12.7]	15 (5%) [78.3±9.3]	30 (10%) [82.5±11.7]
Total no subjects	139 (46.6%) [42.9±21.9]	159 (53.4%) [41.1±19.1]	298 [41.9±20.4]

**Figure 2:**
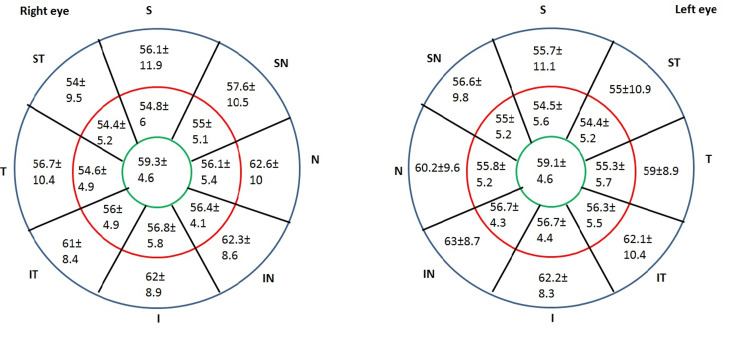
Average corneal epithelial thickness measured by REVO FC spectral-domain optical coherence tomography for 298 Saudi adults (Mean ±SD) in the left eye and the right eye. S: superior; ST: superior temporal; T: temporal; IT: inferior temporal; I: inferior; IN: inferior nasal; N: nasal; SN: superior nasal; SD: standard deviation.

CET measurements taken with the REVO FD OCT instrument for the right and left eyes revealed a high degree of interocular symmetry (ICC = 0.94 (95% CI: 0.937-0.942)). Figure 2 depicts the average CET assessed by REVO FC SD-OCT in the left and right eyes of 298 Saudi adults.

Corneal epithelial thickness (CET) by sector

The CET values for each corneal location are presented in Table 2. The average central CET was 59.2±4.5 µm, while the CET was 7.4 - 8.5% thinner in the paracentral zones of SN (55±5.1 µm), T (54.9±4.9 µm), S (54.6±5.6 µm), ST (54.4±5.2 µm) sectors, and 4 to 6% thinner in the paracentral zones of I (56.7±4.4 µm), IN (56.6±4.1 µm), IT (56.2±4.8 µm) and N (55.8±5.2 µm) sectors than the central CET which was a statistically significant thinning in paracentral zone CETs compared to central CET zone (P˂0.05). Similarly, the midperipheral zones of S (58.5±16.9 µm), and SN (57.6±11.7 µm) sectors showed 1 - 2.7% thinner CETs and ST (54.9±10.9 µm) sector showed 7.5% thinner CET when compared with central CET (P˂0.05). The IN, N, I, and IT sectors were found to be 3.8 - 6% thicker at the midperipheral zones, with average thicknesses of 62.8 ±8.8 µm, 62.2 ±11 µm, 62.1 ±8.3 µm, and 61.5 ±8.4 µm (P˂0.05); whereas the midperipheral T sector showed the similar thickness of central CET (midperiphery T sector (59.8±10.4 µm Vs central CET 59.2±4.5 µm) (P>0.05)). Therefore, the superior and temporal zones were found to have highly thinner CET compared to the inferior and nasal zones, paracentral; the midperipheral inferior and nasal zones were found to be highly thicker than the central CET as well as the midperipheral temporal and superior zones. Also, there is a statistically significant difference in CET values between paracentral and midperipheral zones in all sectors (p˂.05) where the midperipheral zone CET was found to be thicker than the paracentral zone CET, except the ST sector showed no significant change in CET between these two zones (p=0.4751) shown in Table 2.

**Table 2:** Corneal epithelial thickness by section *statistical significance µm ± SD: micrometer ± standard deviation

Section	Paracentral (µm ± SD)	Midperipheral (µm ± SD)	p-value
Nasal	55.8±5.2	62.2±11.0	0.0001*
Inferior nasal	56.5±4.1	62.8±8.8	0.0001*
Inferior	56.7±4.4	62.1±8.3	0.0001*
Inferior temporal	56.2±4.8	61.5±8.4	0.0001*
Temporal	54.9±4.9	59.8±10.4	0.0001*
Superior temporal	54.4±5.2	54.9±10.9	0.4751
Superior	54.6±5.6	58.5±16.9	0.0002*
Superior nasal	55±5.1	57.6±11.7	0.0008*

Correlation of sectorial corneal thickness gender and age

The central CET was found to be 3.5% thicker in males (60.2±4.2 µm) when compared to females (58.1±4.6 µm) which was statistically significant (p˂0.05). Similarly, the paracentral nasal and inferior zones showed 2.2 to 3.6% thicker CET while the superior and temporal paracentral zones showed 3 to 5% thicker CET among males compared to females (p˂.05). Whereas, the midperipheral zone showed 0.5% to 1.7% thicker CET for males which were statistically insignificant (p>0.05). The average CET of males and females and their significance are shown in Table 3. No interocular asymmetry of CT in any sector was noted (p=0.807).

**Table 3:** Average corneal epithelial thickness among males and females *statistical significance

Section	Males (n=139)	Females (n=159)	p-value
Central (µm)	60.2±4.2	58.1±4.6	0.0001*
Paracentral nasal (µm)	56.9±5.0	55.1±5.4	0.0001*
Paracentral inferior nasal (µm)	57.4±4.3	55.6±4.0	0.0001*
Paracentral inferior (µm)	57.3±5.8	56.1±4.4	0.0046*
Paracentral inferior temporal (µm)	57.0±5.2	55.3±5.1	0.0001*
Paracentral temporal (µm)	55.9±4.4	54.0±6.0	0.0001*
Paracentral superior temporal	55.6±4.9	53.2±5.3	0.0001*
Paracentral superior (µm)	56.1±6.4	53.2±4.8	0.0001*
Paracentral superior nasal (µm)	56.0±5.1	54.0±4.9	0.0001*
Midperipheral nasal (µm)	62.1±10.2	62.4±13.8	0.7629
Midperipheral inferior nasal (µm)	64.1±10.2	61.5±7.8	0.0005*
Midperipheral inferior (µm)	62.1±7.6	62.0±9.6	0.8879
Midperipheral inferior temporal(µm)	61.4±8.2	61.8±10.6	0.6066
Midperipheral temporal (µm)	58.3±8.2	58.2±15.8	0.9228
Midperipheral superior temporal (µm)	54.0±7.9	54.4±10.9	0.6082
Midperipheral superior (µm)	57.5±15.8	58.1±19.6	0.6809
Midperipheral superior nasal (µm)	58.1±12.6	57.1±11.8	0.3177

The central CET (2 mm) was 55.4±3.8 µm, 57.2±3.8 µm, 58.3±5.7 µm, 60.4±5.4 µm, 62.2±6.7 µm, 61.2±5.0 µm, and 60.3±4.5 µm for the seven female groups, respectively, demonstrating significant increase in CET with age (r= 0.307; p˂0.05) (Table 4). Group 1 subjects showed significantly thinner CET when compared with groups 4, 5, and 6, and group 2 subjects showed significantly thinner CET when compared with group 5 subjects (p˂0.05) (Table 4). The paracentral I, IT, T, and SN sectors showed a significant increase in CET with increasing age (p˂0.05). For the I, IT, and T sectors, groups 1 and 2 subjects were found to have significantly thin CET compared to group 6 subjects; the SN sector showed a significant increase in CET for group 7 subjects when compared with groups 1, 2, and 3 (p˂0.05), whereas paracentral N, IN, ST, and S sectors showed no difference in CET with the increase in age (p>0.05). In females, the midperipheral sectors showed no difference in CET with age (p>0.05) except the IN sector showed a significant change in CET with the increase in age for group 1 vs group 7; group 3 vs group 5, 6, 7 (p˂0.05). The mean CET with standard deviation for each group with correlation value and significance for females were shown in Table 4.

**Table 4:** Corneal epithelial thickness in different zones in seven groups of females according to age *statistical significance

Section	Age Groups							Correlation With Age	
	10-20 y	21-30 y	31-40 y	41-50 y	51-60 y	61-70 y	˃ 70 y	R	p-value
Central (2 mm)	55.4±3.8	57.2±3.8	58.3±5.7	60.4±5.4	62.2±6.7	61.2±5.0	60.3±4.5	0.307	0.0001*
Paracentral (2-5 mm)									
Paracentral nasal	54.1±5.5	54.6±5.2	54.6±5.3	56.3±4.4	54.7±4.0	59.1±7.6	55.7±3.5	0.014	0.0716
Paracentral inferior nasal	54.6±3.4	55.1±3.7	55.8±4.5	57.0±4.3	57.2±3.1	57.3±6.4	56.0±2.4	0.155	0.0157*
Paracentral inferior	54.6±4.3	55.4±4.1	56.2±4.3	58.1±4.4	57.4±4.5	60.3±5.2	56.0±1.9	0.258	0.010*
Paracentral inferior temporal	53.1±3.2	55.0±5.6	55.7±5.2	57.5±4.4	56.3±4.8	60.1±4.3	55.5±2.3	0.164	0.007*
Paracentral temporal	52.6±5.2	53.5±6.6	53.7±4.5	56.1±4.3	54.4±3.4	60.1±7.6	52.7±3.2	0.199	0.0007*
Paracentral superior temporal	53.1±6.9	52.3±3.8	53.5±5.8	53.2±4.0	55.9±4.2	55.7±3.9	55.2±11.1	0.088	0.1195
Paracentral superior	52.8±5.8	52.4±3.7	52.9±4.7	53.8±3.5	53.9±4.0	53.0±3.4	52.7±2.9	0.058	0.8325
Paracentral superior nasal	53.7±5.6	53.2±3.9	53.3±5.7	53.9±3.2	55.5±3.3	57.6±7.6	59.8±12.0	0.189	0.0035*
Midperipheral (5-7 mm)									
Midperipheral nasal	61.2±8.1	60.9±10.6	60.8±7.9	62.4±8.8	57.5±7.9	63.7±8.07	60.0±10.2	-0.076	0.5679
Midperipheral inferior nasal	63.2±8.6	61.2±7.7	65.3±8.4	61.1±5.5	58.1±2.8	58.2±5.7	56.2±5.3	-0.143	0.0008*
Midperipheral inferior	62.5±7.9	62.4±10.7	62.7±7.8	63.6±9.2	58.7±6.4	59.5±8.5	62.2±10.2	-0.027	0.5871
Midperipheral inferior temporal	62.7±9.6	61.0±9.9	60.5±8.1	62.3±7.9	58.3±4.3	63.2±3.8	59.3±1.4	-0.229	0.4395
Midperipheral temporal	56.9±8.0	55.7±7.1	57.8±8.0	63.3±12.0	55.9±5.9	61.1±8.4	55.7±7.4	0.014	0.060
Midperipheral superior temporal	56.2±12	52.8±10.7	54.0±7.9	57.9±11.4	56.8±9.6	56.3±9.4	60.8±17.4	0.053	0.2757
Midperipheral superior	58.7±17	54.0±10.7	54.0±7.3	60.4±12.1	61.5±13.0	55.1±9.1	59.5±8.4	0.068	0.1026
Midperipheral superior nasal	58.9±9.6	55.7±9.8	56.4±9.9	59.2±10.8	59.6±5.8	58.2±12.2	67.0±19.7	-0.205	0.0561

For males, the central CET was found to be 58.8±3.1 µm, 60±4.3 µm, 60±4.1 µm, 59.9±4.8 µm, 64.8±6 µm, 63.3±2.2 µm, and 56.5±4.9 µm for the seven age groups, respectively, demonstrating significant change with age (r= 0.345; p˂0.05) (Table 5). The group 1, 2, and 3 showed significantly thinner central CET when compared with groups 5, 6, and 7 subjects. The group 1 and 2 subjects showed significant variation in paracentral CET in N, IN, I, and IT sectors when compared with group 5,6, and 7 subjects with age (p˂0.05). The paracentral T, ST, S, and SN sectors did not significantly differ in thickness with an increase in age (p>0.05). There was no significant difference in CET in midperipheral zones of the cornea among different age groups (p>0.05).

**Table 5:** Corneal epithelial thickness in different zones in seven groups of males according to age *statistical significance

Section	Age Groups							Correlation with Age	
	10-20 y	21-30 y	31-40 y	41-50 y	51-60 y	61-70 y	˃ 70 y	R	p-value
Central (2 mm)	58.8±3.1	60.0±4.3	60.0±4.1	59.9±4.8	64.8±6.0	63.3±2.2	56.5±4.9	0.345	0.0000*
Paracentral (2-5 mm)									
Paracentral nasal	55.6±4.1	57.1±5.5	57.2±5.5	56.4±4.5	58.5±3.7	57.0±3.4	52.3±3.4	0.209	0.0057*
Paracentral inferior nasal	56.3±4.5	57.8±4.6	57.3±4.6	56.4±2.4	58.9±3.0	58.4±2.1	53.5±4.4	0.246	0.0027*
Paracentral inferior	55.7±4.6	57.9±5.5	57.1±8.9	57.7±3.7	59.5±4.9	60.1±2.5	52.7±3.3	0.332	0.0021*
Paracentral inferior temporal	55.3±3.9	57.2±5.8	57.6±4.3	58.9±7.0	60.0±5.1	59.5±2.8	52.5±4.9	0.340	0.0003*
Paracentral temporal	54.6±3.5	55.9±4.4	57.1±4.8	56.6±4.7	58.8±6.6	55.9±3.7	56.0±4.7	0.144	0.1435
Paracentral superior temporal	54.4±3.3	55.7±5.4	57.3±3.1	54.9±3.2	56.3±4.9	53.7±3.2	52.3±5.4	0.158	0.0212*
Paracentral superior	54.8±5.7	56.1±6.0	58.5±8.8	55.8±4.6	55.6±6.2	54.1±3.3	54.7±7.3	0.087	0.4652
Paracentral superior nasal	55.3±4.8	56.4±4.9	56.9±6.5	55.7±4.5	56.9±6.1	55.4±3.0	54.7.7±8.0	0.111	0.8522
Midperipheral (5-7 mm)									
Midperipheral nasal	58.3±7.1	62.8±10.8	63.4±10.3	65.9±7.9	63.2±8.3	62.7±13.0	60.5±6.6	0.096	0.2638
Midperipheral inferior nasal	61.5±8.6	65.7±11.2	64.5±8.8	65.5±8.5	60.7±8.4	61.3±3.7	60.2±3.8	-0.128	0.1552
Midperipheral inferior	61.8±7.3	62.7±8.4	64.2±7.4	61.8±5.7	57.7±3.9	61.9±5.1	61.8±6.0	-0.073	0.21
Midperipheral inferior temporal	60.6±7.7	62.5±8.6	64.9±9.1	61.6±5.7	58.6±2.6	60.0±5.3	59.5±4.5	-0.084	0.1025
Midperipheral temporal	58.1±5.8	58.5±8.9	60.0±9.9	59.9±6.7	59.4±8.6	58.8±5.9	55.7±3.5	-0.114	0.0978
Midperipheral superior temporal	53.4±7.2	54.2±8.2	57.0±9.2	54.6±4.7	51.9±4.3	53.2±9.0	53.7±2.5	-0.024	0.7219
Midperipheral superior	54.7±9.0	55.7±9.5	58.3±9.7	56.9±13.7	59.1±19.0	54.6±6.1	62.5±15.5	-0.033	0.4584
Midperipheral superior nasal	55.7±10.2	57.1±9.2	60.6±10.5	59.0±10.8	58.7±9.5	58.2±5.1	56.2±3.5	-0.04	0.4891

**Figure 3:**
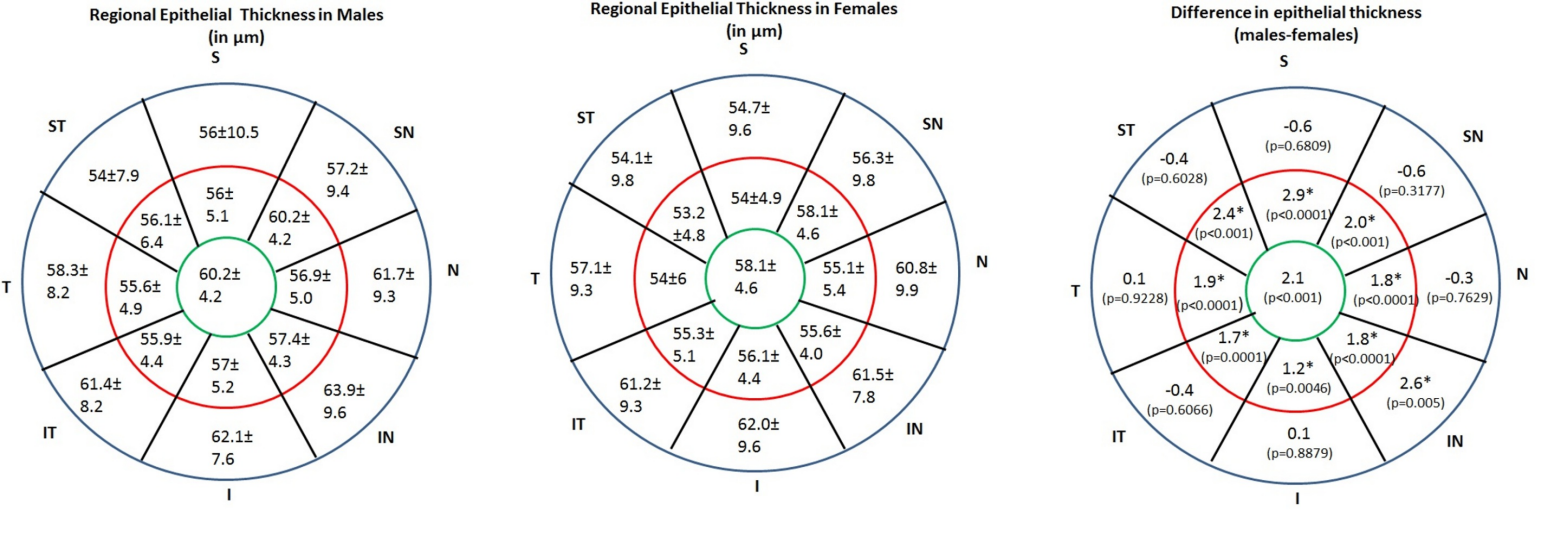
Sector-wise CET distribution by gender [mean (± standard deviation)] and difference in CET in 17 sectors by gender and its significance levels (p). Sectors showing statistically significant differences are shown with an asterisk symbol.

The association between age and regional CET is not very strong, despite a statistically significant positive connection (Tables 4-5). The representative mean (± standard deviation) total CET maps of 17 sectors of males (Left) and females (Middle) are given in Figure 3, along with the significant levels (p) (Figure 3; Right) that were determined by assessing the mean CET of each sector for both genders across the seven age groups.

## Discussion

Establishing a normative database of epithelial thickness profiles using OCT imaging is essential to improving the diagnosis of corneal diseases since recent advancements in OCT allow the measurement of regional CET and its characterization in response to changes in corneal structure. Precise CT and CET measurements are becoming increasingly important in clinical practice since they are necessary for diagnosing ectasia, monitoring the disease’s progression, and planning refractive surgery [18]. To accurately determine the range of normal variation of CET in the Saudi population, this study assessed healthy Saudis’ normative epithelial thickness profile using SD-AS-OCT 7-mm wide scans.

Mean CET across ethnic groups

Various imaging equipment such as AS-OCT, confocal microscopy, and VHF digital ultrasonography have been used to measure central CET, which ranged from 48.0 ± 5 to 59.9 ± 5.9 μm [7,19-22]. In our study, the mean CET in the central 2-mm zone was 59.2±4.5 µm in Saudi Arabian participants. This was consistent with the study performed on normal healthy subjects grouped by age, in which the average central CET was reported to be 60.48 ± 8.37 µm in north Indians using the same instrument (SD-OCT device, Revo Nx, Optopol Technology, Poland) [23]. Similarly, Wang et al. (2004) showed a central CET of 59.9 ± 5.9 μm in subjects aged 39.9 ± 8.6 years using a real-time OCT [24]. The CET values obtained from the present study were greater than those from studies conducted in Egypt (49.57 ± 2.11 μm using Zeiss, Cirrus 5000-OST) [10], Syria (50.70 ± 3.56 μm using Zeiss, Cirrus 5000-OST) [25], Jordan (53.7 ± 4.0 μm using Optovue Avanti SD-OCT) [26], and Turkey (47.88±1.15μm using ultrasound) [9]. The average central CET values for the right and left eyes in this study were 59.3±4.6 µm and 59.1±4.6 µm, respectively. Using the 9-mm wide AS-OCT scan, the central CET values for the right and left eyes in the Indian population were reported to be 54.37 ± 3.75 μm and 54.13 ± 4.51 μm, respectively [27]. These values were lower than the central CET values of the right and left eye in the present study. People differ from one another due to hereditary differences, genetic differences, and differences in environmental exposure. Alsaqr et al. (2021) investigated the corneal parameters of Saudi Arabians and evaluated the differences in corneal topography between Arabs and other ethnicities, demonstrating that Saudi Arabians have unique corneal characteristics that differ from the different ethnic groups. Saudi Arabians had a wider palpebral aperture, greater horizontal visible iris diameter, and prolate corneas than Caucasians, Chinese, and Japanese individuals. This may help to explain why Saudi Arabians have a higher prevalence of dry eye and keratoconus, especially given the hot, dry climate [28]. Given that the CE is an extension of the skin's epidermal layer, it is reasonable to assume that individuals who live in hotter climates and have been exposed to intense sunlight would have thicker corneas with darker irises and thicker skin with higher melanin pigmentation [29].

Regional variations of CET

Studies on epithelial mapping showed that the corneal zones’ CET profiles were non-uniform and differed in thickness [7,15,30-32] with temporal zones thinner than nasal zones and superior zones thinner than inferior zones [1,7,15,30-37]. Our study found a similar pattern of nonuniformity, with CET being significantly thinner superiorly than inferiorly (paracentral zone S: 54.6±5.6 µm Vs I: 56.7±4.4 µm; paracentral ST: 54.4±5.2 µm Vs IT: 56.2±4.8 µm, paracentral SN: 55±5.1 µm Vs IN: 56.5±4.1 µm; midperipheral zone S: 58.5±16.9 µm Vs I: 62.1±8.3 µm; midperipheral ST: 54.9±10.9 µm). Additionally, the midperipheral N and T sectors were 8.5% and 10.8% thicker than the paracentral N and T sectors, respectively. The midperipheral I, IN, and IT sectors were 9 to 10.5% thicker, while the S and SN sectors were 4.6 to 6.9% thicker than their respective paracentral sectors. Overall, regional CET patterns were comparable to prior results, with the superior and temporal epithelial thickness being lower than inferior and nasal epithelial thickness [36]. The dynamics of the upper eyelid are responsible for the superior segment’s lower CET values. In 1994, it was shown that blinking and friction on the cornea might control the CET profile [37]. Numerous investigations have demonstrated the dynamics of the anatomy of the eyelids during blinking, wherein the upper lid’s vertical traverse is noticeably larger than that of the lower lid and the upper lid falls at its fastest point when it crosses the visual axis, potentially causing surface epithelium chafing. This, in turn, places more stress on the superior than the inferior cornea [1,37,38]. This could explain the superiorly observed increased epithelial thinning. The stromal thickness showed the notable opposite regional variations reported by Kim et al. (2020) [36], suggesting that the superior stroma is thicker than the inferior stroma. Since CE nonuniformity may be crucial to establishing and maintaining a smooth, symmetrical optical surface, it masks the presence of altered regional variations in stromal thickness [37,38].

Gender differences in CET

The central CET was higher in males (60.2±4.2 µm) than in females (58.1±4.6 µm). The central CET of male subjects was found to be 2.1µm thicker than that of female subjects. Additionally, it was observed that the paracentral zones of CET in males were much thicker than those in females, although the midperipheral zones did not differ significantly in thickness. According to Wu and Wang (2017) [32], males’ central and ring 2 zone CETs were 1.39 μm thicker than those of females. Furthermore, Hashmani et al., (2018) [28], Kanellopoulos and Asimellis (2013) [12], and Wu and Wang (2017) [32] found that the central CET of male and female subjects differed by 1.9 μm, 1.52 μm, and 1.34 μm. According to research by Kim et al. (2016) [15], men had higher CETs than women, and these gender disparities should be considered when evaluating CET. Gender influences CET in healthy individuals, which may be related to the endocrine variations between men and women [39-42]. Human ocular tissues, including the cornea, iris, ciliary body, lens, conjunctiva, lacrimal sac, and meibomian glands, have been shown to contain gonadal hormone receptors (Kazama et al., 2019) [40]. The cornea’s epithelium, stroma, and endothelium are hormone-responsive, gender-dependent tissues that contain sex hormone receptors. Changes in female sex hormone levels may directly impact corneal thickness, as corneal epithelial cells contain androgen, progesterone, and estrogen receptors [43,44]. Studies have revealed that estrogen receptors in the human cornea play a role in corneal physiology by demonstrating a considerable variation in corneal hydration during the normal menstrual cycle, and these changes could be associated with the actions of estrogen rather than progesterone [45]. Specifically, estrogen receptors in human corneas cause the cornea to thicken at the end of the menstrual cycle and thin at the beginning, indicating that estrogen might be involved in determining corneal thickness. As a result, the central CT varies throughout the menstrual cycle [46]. This shows that estrogen may alter corneal physiology, as well as CT and CET. Interestingly, the gender difference was higher for epithelial thickness than stromal thickness. Thus, to improve clinical risk assessment during the preoperative evaluation for laser refractive surgery and glaucoma management, as well as to tailor interventions and procedures more accurately, ophthalmic specialists must comprehend the physiological effects of female sex hormones on corneal structure and function, as well as the ocular symptoms that females experience as they age [45,46]. Therefore, our findings support that gonadal hormones may affect ocular tissue growth.

Effects of aging on CET

Corneal ET of a central 2-mm diameter zone in groups 1, 2, 3, 4, 5, 6, and 7 were 57.1±3.5 µm, 58.6±4.1 µm, 59.1±4.9 µm, 60.1±5.1 µm, 63.5±6.4 µm, 62.2±3.6 µm, and 58.4±4.7 µm respectively, showing significant increase in thickness with aging (from group 2 to 6). The CET in group 1 was thinner than the adults’ groups (21-70 years). However, several studies found no association between CET and age [1,47], whereas Yang et al. (2015) [14] and Kim et al. (2016) [15] found that the CET thins in the paracentral and midperiphery with age, but the central 2-mm diameter zone remains stable. Also, these studies have shown that the CE of children was thinner than that of young adults (18-29 years) and thicker than the old that of old adults (60-80 years) in the paracentral and midperipheral areas however the central 2-mm diameter zone remains stable [14,15]. Considering our findings and the results of previous studies, the CE seems to become thicker during childhood, and remain stable during early adulthood but become thinner in old age, especially in the periphery. During normal homeostasis, limbal epithelial stem cells replenish and maintain the CE. According to Zheng and Xu, these limbal epithelial stem cells exhibited quantity decline and age-related size enlargement with increasing age. These results implied that the ability of limbal epithelial stem cells to proliferate decreases with age, leading to a reduction in their ability to sustain the stability and integrity of the CE. This could explain our observations that the CE of the elderly was thinner than that of the young [48].

## Conclusions

This study provides the first comprehensive characterization of CET profiles in the normal Saudi subjects, filling a gap in the regional literature. Age and gender have shown their effect on CET of the central 2-mm zone being thicker with increasing age, and CET was found to be thicker in males. The CET of the left and right eyes did not differ from one another. Furthermore, the CET indicated substantial difference between the superior and inferior medians, with the superior epithelium being the thinnest. These findings could be valuable in deciding corneal refractive surgeries, evaluating corneal ectatic conditions, and reducing the risk of refractive surprises in Trans-epithelial photorefractive keratectomy. This is the first study to evaluate the features of CET in normal participants, which will be a useful addition to the literature on normal ocular parameters in the Middle East area. It also illustrates the contrasts seen with normative CET data collected globally. Additional research with larger sample sizes and limbal CET in normal corneas is required which in turn provide a foundation for studying ocular surface pathology. There are several limitations in the current study. Firstly, the main limitations were the CET was measured using a single OCT device, and the sample distribution was not equal among the age group categories. Secondly, since the precorneal tear film, which was estimated to be 4.79 µm, is included in the OCT measurement of CET, changes to the tear layer may affect CET measurement. We hypothesize that the rate of tear film thinning rises with aging as tear breakup time decreases. This would cause the tear-film thickness to decrease, confounding the current study’s findings.
